# Diabetic testicular dysfunction and spermatogenesis impairment: mechanisms and therapeutic prospects

**DOI:** 10.3389/fendo.2025.1653975

**Published:** 2025-08-25

**Authors:** Wenxiu Zhang, Li Tong, Baofang Jin, Dalin Sun

**Affiliations:** ^1^ Department of Traditional Chinese Medicine, Qinghai Unversity Medical College, Xining, Qinghai, China; ^2^ Andrology Department of Integrative Medicine, Zhongda Hospital, School of Medicine, Southeast University, Nanjing, Jiangsu, China

**Keywords:** diabetes mellitus, oxidative stress, AGEs, testicular damage, spermatogenesis impairment

## Abstract

With the global prevalence of diabetes mellitus (DM) steadily increasing, its impact on male reproductive health has become a growing area of concern. Diabetes-induced testicular damage involves alterations in testicular cell function, hormone levels, and the integrity of the blood-testis barrier (BTB), ultimately disrupting spermatogenesis. The key pathogenic factors include hyperglycemia, oxidative stress, chronic inflammation, mitochondrial dysfunction, and the accumulation of advanced glycation end products (AGEs).This review synthesizes the latest research on diabetes-induced testicular dysfunction and spermatogenic impairment, while also exploring potential therapeutic strategies. Current interventions are primarily focused on glycemic control, with supplementary treatments involving Chinese medicine, nanoparticles, and probiotics. Although most of the current evidence is derived from preclinical studies, these findings provide important insights that may inform future clinical research on diabetes-related male reproductive dysfunction.

## 1 Introduction

Diabetes mellitus (DM) is a chronic metabolic disorder that includes both type 1 diabetes mellitus (T1DM) and type 2 diabetes mellitus (T2DM) (1). T1DM is characterized by absolute insulin deficiency resulting from autoimmune destruction of pancreatic β-cells, whereas T2DM primarily results from insulin resistance, commonly associated with overweight, obesity, and unhealthy lifestyle factors (2). In recent years, the global prevalence of diabetes has continued to rise, with T2DM accounting for the majority of cases (3, 4). By 2045, it is projected that the global number of individuals with diabetes will reach 693 million. Notably, the prevalence of diabetes is consistently higher in men than in women across all age groups (5). The pathogenesis of diabetes involves a complex interplay of multiple factors, including hyperglycemia, oxidative stress, chronic inflammation, mitochondrial dysfunction, and endoplasmic reticulum (ER) stress, all of which contribute to multi-organ complications (6, 7). In addition to the well-established microvascular and macrovascular complications, mounting evidence suggests that diabetes also negatively impacts male reproductive health.

Epidemiological data suggest that approximately 15% of couples worldwide experience infertility, with male factors contributing to 40–50% of cases. Diabetes has been linked to an increased incidence of male infertility. Recent clinical and preclinical studies have demonstrated that diabetes induces widespread damage to the male reproductive system (7, 8). Diabetic testicular dysfunction is primarily characterized by impaired spermatogenesis and disruption of the sperm microenvironment, including altered testosterone secretion and structural damage to testicular tissue. These alterations are mainly associated with metabolic dysregulation of Sertoli cells, increased apoptosis of Leydig cells, and compromised integrity of the blood-testis barrier (BTB) (9, 10).

While glycemic control remains the cornerstone of diabetes management, increasing attention is being given to exploring therapeutic interventions aimed at mitigating diabetes-induced damage to the reproductive system. This review aims to summarize the current research progress, elucidate underlying mechanisms, assess the clinical applicability of emerging treatments, and explore potential strategies that may inform future clinical interventions.

## 2 Sperm damage

DM exerts a profoundly detrimental effect on male reproductive function (2). Both T1DM and T2DM have been shown to impair spermatogenesis, characterized by reduced semen volume, sperm count, concentration, and motility, along with increased DNA fragmentation and abnormal morphology (11–15). Notably, T1DM appears to more significantly impair spermatozoa progressive motility and is associated with a higher rate of semen anti‐sperm antibody positivity, whereas T2DM is more strongly linked to decreased sperm concentration and an increased proportion of late apoptotic spermatozoa (16). The underlying mechanisms include chronic inflammation, oxidative stress, mitochondrial dysfunction, and ER stress. Additionally, T1DM may be associated with a lack of physiological contraction of the cranial and caudal portion of the epididymis after ejaculation (17, 18). However, the mechanisms underlying hormonal disturbances, gonadal dysfunction, and male accessory gland infection (MAGI) remain incompletely understood (15). Spermatogenesis is highly dependent on metabolic support from SC, which utilize glucose as a primary energy substrate. Glucose transporters (GLUTs) facilitate the uptake of extracellular glucose into sertoli cell (SC), where it is converted into pyruvate and subsequently reduced to lactate by lactate dehydrogenase (LDH). Lactate is then exported via monocarboxylate transporters (MCTs) to developing germ cells, serving as their primary energy source. DM has been shown to significantly disrupt the expression of key components in this metabolism pathway, leading to decreased lactate availability and energy supply to germ cells (19–22). This metabolic disturbance contributes to ejaculatory dysfunction (including anejaculation, retrograde ejaculation, and asthenozoospermia) and erectile dysfunction (16). Furthermore, T2DM is frequently accompanied by metabolic syndrome and cardiovascular disease, compounding its negative effects on male reproductive health (18) (**Table 1**).

**Table 1:** Differential pathological mechanisms and sperm damage profiles in type 1 and type 2 diabetes.

Feature	T1DM	T2DM
Pathological mechanism	Destruction of pancreatic β‐cells, either by autoimmune or idiopathic, absolute insulin deficiency	Insulin resistance
Important pathological factors	Hyperglycemia and insulin deficiency	Hyperglycemia and insulin deficiency Overweight and obesity Unhealthy life styles
Potential mechanisms	Inflammation Oxidative stress Mitochondrial dysfunction Endoplasmic reticulum stress Lack of physiological contraction of the cranial and caudal portion of the epididymis after ejaculation	Hyperglycemia Dyslipidemia Inflammation Amicrobial inflammatory Increased concentration of seminal fluid leukocytes Oxidative stress Mitochondrial dysfunction Endoplasmic reticulum stress
Semen parameters	Spermatozoa progressive motility↑ Semen volume↓ Total sperm count↓ Sperm motility↓ Abnormal sperm morphology↓ Rate of semen anti‐sperm antibody positivity↓ Sperm DNA fragmentation↑	Sperm concentration↓ Semen volume↓ Total sperm count↓ Sperm motility↓ Abnormal sperm morphology↓ Sperm DNA fragmentation↑ Proportion of late stage apoptotic sperm↑
Complications	Ejaculation disorders(Anejaculation,Retrograde ejaculation,Asthenozoospermia) Erectile dysfunction	Metabolic syndrome Cardiovascular diseases Ejaculation disorders(Anejaculation,Retrograde ejaculation,Asthenozoospermia) Erectile dysfunction

↓, decreased; ↑, increased.

### 2.1 Sperm DNA damage and abnormal sperm morphology

Chronic hyperglycemia and insulin resistance lead to systemic immune dysregulation and sustained inflammation within the male reproductive tract (23). This state of inflammation, characterized by abnormal activation of leukocytes, leads to an overproduction of reactive oxygen species (ROS). These ROS oxidize protamines, crucial for the correct condensation of sperm nuclei, leading to the disruption of disulfide bond formation. Consequently, incomplete chromatin packaging occurs, leading to heightened DNA fragility and genomic instability (24). These nuclear abnormalities are closely associated with morphological defects, particularly affecting the sperm head (25). Additionally, elevated levels of advanced glycation end products (AGEs) and DNA fragmentation markers have been consistently detected in the sperm of diabetic men, indicating substantial nuclear instability and chromatin disruption (26). Prolonged hyperglycemia disrupts normal spermatogenesis by interfering with the development of key structural components, including the sperm head, midpiece, and tail, leading to a significant increase in morphologically abnormal sperm (27).

### 2.2 Reduction in sperm count

DM, particularly T2DM, significantly reduces sperm count through multiple interrelated mechanisms. It disrupts the hypothalamic-pituitary-gonadal (HPG) axis, resulting in decreased levels of testosterone, follicle-stimulating hormone (FSH), and luteinizing hormone (LH) (28). The reduction in hormone levels directly impairs critical stages of spermatogenesis, particularly the primary meiotic division, and disrupts the function of both SC and Leydig cell(LC). A reduction in the number of SC, combined with impaired lactate production, leads to an inadequate energy supply for germ cells, thereby promoting germ cell death (29, 30). Furthermore, oxidative stress activates apoptotic signaling pathways—such as the MAPK/p38 pathway— upregulating the expression of the pro-apoptotic protein Bax while downregulating the anti-apoptotic protein Bcl-2. This dysregulation ultimately triggers extensive programmed cell death among spermatogenic cells, including SC and LC (24). Structural and functional defects in the sperm tail further impair sperm motility and viability. Ultimately, hormonal imbalances, SC dysfunction, oxidative stress, defective chromatin maturation, excessive activation of apoptotic pathways, and abnormalities in sperm structure and function collectively lead to severe disruption of spermatogenesis. This results in a substantial decline in daily sperm production, sperm reserve quantity, and impaired overall testicular spermatogenic function (27, 31).

### 2.3 Decreased sperm motility

DM impairs sperm motility through a complex interplay of hormonal imbalance, oxidative stress, metabolic dysfunction, and inflammation (23, 27, 32). It compromises testicular antioxidant capacity and promotes germ cell apoptosis, leading to reduced testosterone levels. This testosterone deficiency disrupts epididymal function, weakens reproductive antioxidant defenses, and alters the expression of proteins critical for sperm flagellar movement and acrosome integrity, ultimately diminishing sperm motility (27, 33).

Trace element imbalances also contribute to diabetic testicular dysfunction. Ghasemi et al. observed significantly reduced concentrations of zinc and magnesium in the seminal plasma of diabetic individuals. Zinc levels exhibited a strong positive correlation with sperm motility and morphology, whereas magnesium was significantly associated with parameters of sperm motility (34).

Inflammation is another important factor. The accumulation of AGEs activates macrophages through the receptor for AGEs (RAGE), triggering the release of pro-inflammatory cytokines such as tumor necrosis factor-α (TNF-α). *In vitro* studies have demonstrated that TNF-α can directly suppress sperm motility by 40%–60% (35, 36).

Furthermore, sperm motility is highly dependent on energy derived from glucose metabolism. Insulin deficiency or resistance interferes with glucose utilization, limiting energy availability for sperm motility (29).

## 3 Mechanisms of spermatogenesis disorders

### 3.1 Mitochondria and oxidative stress

Mitochondria play a pivotal role in regulating sperm function by modulating essential processes, including metabolism, signal transduction, energy production, and responses to oxidative stress. The midpiece of sperm is rich in mitochondria, which generate ATP through the electron transport chain (ETC), providing the energy required for sperm motility, capacitation, and overall functional capability (37). Additionally, mitochondria are essential for maintaining DNA integrity, regulating calcium homeostasis, mediating apoptotic signaling, and supporting the differentiation and maturation of spermatogenic cells (38). As the primary cellular energy generators, mitochondria are also the main source of ROS within the cell. Physiological levels of ROS are essential for sperm capacitation and fertilization. The antioxidant defense systems in semen generally maintain redox homeostasis under normal conditions. However, excessive ROS production can inhibit the activity of key antioxidant enzymes such as superoxide dismutase (SOD) and glutathione peroxidase (GPx), leading to oxidative stress and mitochondrial dysfunction. Therefore, maintaining mitochondrial integrity is crucial for preserving sperm quality, and its impairment is widely recognized as a major cause of male infertility (39–41).

### 3.2 Hypothalamic-pituitary-gonad axis function

Insulin regulates reproductive function in both males and females by modulating the HPG axis. This effect is probably mediated by the MAPK/Erk1/2 pathway, whereby insulin stimulates the hypothalamus to secrete gonadotropin-releasing hormone (GnRH) (42). GnRH subsequently acts on the pituitary gland to promote the synthesis and release of LH and FSH, which in turn facilitate the maturation of seminiferous tubules and LC (2). Insulin resistance disrupts the function of the HPG axis, directly leading to decreased secretion of LH and FSH, reduced testosterone levels, and an imbalance in the androgen-to-estrogen ratio (43, 44). Meanwhile, persistent hyperglycemia further exacerbates reproductive dysfunction by impairing the pulsatile release of GnRH from the hypothalamus and suppressing LH and FSH synthesis in the pituitary. These disturbances ultimately impair testosterone production by LC and compromise the supportive function of SC in spermatogenesis, resulting in impaired spermatogenic function (2).

### 3.3 Advanced glycation end products

AGEs can severely impair sperm function through multiple mechanisms. Upon binding to RAGE, AGEs not only activate inflammatory signaling pathways but also directly disrupt key cellular processes and structural components of sperm. Notably, AGEs exposure significantly reduces sperm motility, particularly progressive motility (45, 46). This decline may result from AGEs-induced mitochondrial dysfunction, crosslinking with membrane proteins or flagellar components, and the induction of DNA damage and activation of apoptotic pathways. In addition, AGEs–RAGE interactions may interfere with essential fertilization-related processes such as capacitation and the acrosome reaction, further compromising the fertilizing potential of sperm (35, 37).

## 4 Damage to the environment surrounding spermatogenesis

### 4.1 Sertoli cell

The number of SC in the adult testis determines daily sperm production, as each SC can maintain a limited number of germ cells (47). SC play a critical role in forming the BTB and providing nutrients essential for spermatogenesis. SC can metabolize a variety of substances, including glucose, lactic acid, fatty acids, and amino acids, and possess specific glucose sensing mechanisms that are highly sensitive to extracellular glucose levels (48). The glucose transporters GLUT1 and GLUT3 work synergistically in SC to maintain glucose uptake and ensure lactate production. Under diabetic conditions, the metabolic homeostasis of SC is disrupted, potentially leading to structural and functional impairment of the BTB in patients with T2DM (49). Both *in vivo* and *in vitro* studies have demonstrated that high glucose levels upregulate Apelin (APLN) in SC, which subsequently downregulates cell junction–related genes such as connexin 43 (Cx43) and zonula occludens-1 (ZO-1), ultimately compromising the integrity of the BTB (50).

### 4.2 Increased permeability of blood-testis barrier

The blood–testis barrier (BTB), also referred to as the SC barrier, is a critical structural and functional component of the seminiferous tubules. Located at the basal compartment of the seminiferous epithelium, the BTB is primarily formed by junctional complexes between SC, including tight junctions, gap junctions, and basal ectoplasmic specializations (51). The primary function of the BTB is to segregate the lumen of the seminiferous tubules from the systemic circulation, thereby establishing an immune-privileged microenvironment that protects developing germ cells from harmful substances and maintains the sterile conditions essential for spermatogenesis (52, 53).

During spermatogenesis, spermatogonia migrate across the BTB within the seminiferous tubules, the functional units of the mammalian testis. To facilitate this translocation, cell junctions are dynamically disassembled and reassembled, enabling the movement of immature germ cells from the basal compartment to the luminal compartment as they undergo meiosis and subsequent differentiation, ultimately culminating in the release of mature spermatozoa (54, 55). This process involves proteases, protease inhibitors, and cell-junction components (56).

The interaction between Cx43 and ZO-1 is crucial for the maintenance of BTB integrity. Hyperglycemia and AGEs have been shown to impair the expression and function of junctional proteins such as Cx43 and ZO-1 in SC by inducing oxidative stress, inflammation, and hormonal disturbances, ultimately compromising BTB function. Genetic deletion of Cx43 leads to BTB disruption and subsequent failure of spermatogenesis (11). Ke Song’s STRT-seq analysis of testicular tissue from diabetic patients at single-cell resolution revealed significant alterations in SC gene expression profiles and disruption of the BTB structure. *In vivo* biotin tracer assays in hyperglycemic (HGM) mice demonstrated a substantial increase in biotin-positive seminiferous tubules and deeper biotin penetration, indicating a loss of BTB integrity induced by hyperglycemia (50).

### 4.3 Alterations in Leydig cell secretory function

Basel A. Abdel-Wahab et al. reported histopathological changes in diabetic rat testes, including seminiferous tubule atrophy and significant degeneration and necrosis of LC (57).LC located in the testicular stroma, are regulated by LH to produce testosterone, essential for maintaining spermatogenesis. Androgens have a bidirectional relationship with glucose regulation (58). Androgen deficiency is a risk factor for T2DM, while hyperglycemia promotes the formation of AGEs through non-enzymatic glycation. These AGEs bind to receptors such as RAGE in the testes, triggering overactivation of downstream signaling pathways that downregulate key steroidogenic enzymes, ultimately impairing testosterone secretion (59, 60). Furthermore, chronic hyperglycemia itself induces ER stress within LC. This ER stress disrupts LC function, causing cell cycle arrest, apoptosis, and further suppression of testosterone synthesis. Collectively, these mechanisms disrupt the testicular reproductive environment and impair sperm production, contributing to male reproductive dysfunction in diabetes (59, 61, 62). Insulin resistance further exacerbates this by reducing insulin sensitivity and triggering compensatory hyperinsulinemia, which promotes facilitates the conversion of testosterone to dihydrotestosterone (DHT) and suppresses LH and FSH secretion, thereby disrupting the synthesis and regulation of reproductive hormones (63, 64). Consequently, men with T2DM frequently present with hypogonadism, which is closely associated with early LC dysfunction.

## 5 Therapeutic strategies for diabetes testicular dysfunction

### 5.1 Antidiabetic agents treatments

Current therapeutic strategies for diabetes-associated testicular dysfunction remain limited, with a predominant emphasis on glycemic control (**Table 2**). Studies have demonstrated that metformin can improve sperm quality, an effect attributed to its ability to elevate testosterone levels and support spermatogenesis. This beneficial action is closely linked to the restoration of gonadotropic hormone and leptin system function within the testes. These effects are largely attributed to improved insulin sensitivity, which contributes to hormonal balance and promotes testicular function and integrity (65, 66). Conversely, evidence suggests that the use of sulfonylureas and thiazolidinediones may be associated with impaired sperm quality, exerting detrimental effects on motility and vitality (67). Emerging evidence suggests that semaglutide, a glucagon-like peptide-1 receptor agonist (GLP-1 RA), may offer a more comprehensive therapeutic approach for male infertility associated with diabetes and obesity. In diabetic rat models, semaglutide has been shown to restore redox homeostasis, modulate androgen levels, attenuate testicular inflammation, and alleviate diabetes-induced testicular damage by regulating the ferroptosis pathway (68). Furthermore, semaglutide improves the expression of key genes and proteins involved in spermatogenesis through multiple molecular mechanisms, thereby preventing testicular and sexual dysfunction induced by diabetes (69). In a 24-week clinical trial, semaglutide significantly improved sperm morphology in men with type 2 diabetes, obesity, and functional hypogonadism. Notably, its effects on sperm concentration and total sperm count were superior to those achieved with testosterone replacement therapy (TRT), offering a novel therapeutic option for this patient population (30). Liraglutide improves energy homeostasis by promoting fatty acid utilization in spermatogonia, thereby enhancing the energetic state of seminiferous tubules. Additionally, it exerts anti-inflammatory and antioxidant effects, contributing to the improvement of sperm quality and hormonal profiles (70, 71). A 16-week study demonstrated that liraglutide effectively facilitates weight loss, ameliorates sexual function, elevates testosterone levels, and restores HPT axis function. Furthermore, liraglutide positively impacts metabolic health by improving glycemic control, reducing HbA1c, alleviating insulin resistance, and mitigating metabolic syndrome (72). Simultaneously, another study investigating liraglutide treatment in obese men with metabolic hypogonadism reported significant improvements in sperm motility, semen parameters, and sexual function after four months of therapy, compared to both gonadotropin and transdermal testosterone groups. Moreover, increases in testosterone, sex hormone-binding globulin (SHBG), and gonadotropin levels were also observed (73). The GLP-1 peptide-based agents discussed above have demonstrated the potential to enhance spermatogenesis and testosterone synthesis in both preclinical and clinical studies, offering valuable insights for the development of therapeutic strategies in clinical practice. Consequently, addressing testicular dysfunction and abnormalities in spermatogenesis continues to represent a meaningful and promising direction for future research.

**Table 2:** Therapeutics in diabetes-induced testicular damage.

Therapeutic category	Specific intervention	Core protective mechanism	References
Antidiabetic Agents	GLP-1 RA: • Semaglutide • Liraglutide	Restores redox homeostasis Modulates androgen levels Attenuates testicular inflammation Regulates ferroptosis (Semaglutide) Promotes fatty acid utilization in spermatogonia (Liraglutide)	(30, 68–73)
	Metformin	Enhances insulin sensitivity Increases testosterone Supports spermatogenesis	(65, 66)
	Sulfonylureas/Thiazolidinediones	Impairs sperm motility/vitality (adverse effect)	(67)
Probiotics	Bifidobacterium longum	Antioxidant, anti-apoptotic, regulates sex hormones	(74)
	Lactobacillus casei + ω-3 fortified soy milk	Increases Sertoli/Leydig cell count	(75)
	Lactobacillus	Synergistically modulates gut microbiota, inhibits oxidative stress	(76)
	Probiotic/Synbiotic therapy	Modulates gut microbiota Reduces oxidative stress Improves sperm DNA integrity/concentration/motility	(77–80)
Chinese Medicine	Plant compounds: Stevioside, Tripterine, Silymarin, Phloridzin, Icariin, Dognoside, Aucubin, Dendrobium polysaccharides, Propolis	Inhibits oxidative stress/inflammation (NF-κB, AGEs/RAGE/Nox4) Enhances antioxidant defenses (Nrf2, Hsp70/90) Improves BTB integrity (ZO-1) Modulates gut microbiota (Phloridzin/Dognoside)	(11, 12, 14, 62, 81–87)
Nanotechnology	Selenium Nanoparticles (SeNPs)	Suppresses redox imbalance Upregulates Nrf2 Improves sperm quality Targeted drug delivery	(90, 91),
	Zinc Oxide Nanoparticles (ZnO NPs)	Antioxidant, hypoglycemic, downregulates pro-apoptotic genes, upregulates steroidogenic genes	(92, 93),
Other Therapies	Montelukast	Anti-inflammatory, antioxidant, activates autophagy	(94)
	Bezafibrate	Suppresses inflammation and oxidative stress	(95)
	Tropisetron	Alleviates testicular inflammation	(96)
	Ranolazine, Rapamycin, Chromium Picolinate	Induces autophagy, inhibits ER stress, antioxidant, anti-apoptotic	(97–99)
	Islet Transplantation (IT)	Delays testicular interstitial fibrosis	(100, 101)
Combination Therapies	Zinc + Metformin	Activates PI3K/AKT/mTOR pathway, corrects zinc homeostasis	(102)
	Vitamin D + Metformin	Synergistically enhances improvement of testicular dysfunction	(103)
	Chrysin + MIRET	Reduces pro-apoptotic effects	(104)
	Cilostazol + Sildenafil	Anti-inflammatory, antioxidant	(105)
	Sitagliptin + L-Theanine	Synergistically mitigates testicular injury	(106)

### 5.2 Probiotic treatments

Bifidobacterium longum exerts multifaceted anti-diabetic effects, with its antioxidant and anti-apoptotic properties contributing to improvements in reproductive function and the regulation of hormone levels (74). Soy milk fortified with Lactobacillus casei and omega-3 inhibits the infertility phenotype in a rat model of type 1 diabetes, increasing the number of SC and LC and enhancing sperm quality (75). The combination of Lactobacillus, montelukast, and metformin synergistically mitigates diabetes-induced testicular damage by modulating the intestinal microbiome and reducing oxidative stress (76).Multiple randomized controlled trials (RCTs) have demonstrated that probiotic supplementation significantly improves sperm parameters in infertile men, including sperm concentration, motility, and morphology. In addition, a marked reduction in oxidative stress biomarkers has been reported, suggesting that probiotics help restore redox homeostasis within the seminal microenvironment (77–79). Probiotics may also influence male hormonal profiles. Several studies have reported a mild increase in serum testosterone levels and improved hormonal balance following probiotic supplementation, which may support spermatogenesis. Furthermore, synbiotic therapy (a combination of probiotics and prebiotics) has shown beneficial effects in certain trials, including improvements in sperm DNA integrity and chromatin quality (80). Lactobacillus and Bifidobacterium species are among the most commonly used and well-studied probiotic strains. These bacteria can lower intestinal pH and significantly enhance sperm quality through antioxidant, anti-inflammatory, and gut microbiota-modulating mechanisms. Clinically, probiotic-based treatments are low-cost, safe, and offer promising potential as adjunctive therapies for improving sperm quality in men with diabetes. Nevertheless, large-scale clinical studies are still needed to determine the most effective strain combinations and to validate their long-term efficacy (77).

### 5.3 Chinese medicine treatments

A variety of plant-derived compounds, including stevioside, tripterine, silymarin, phloridzin, icariin, dognoside, Aucubin, dendrobium polysaccharides, and propolis, have demonstrated protective effects against testicular damage induced by diabetes or inflammation (11, 12, 14, 62, 81–87). The core mechanisms underlying these protective effects involve inhibition of oxidative stress and inflammatory pathways (e.g., regulation of NF-κB, Nrf2, AGEs/RAGE/Nox4), reduction of apoptosis and necrosis, enhancement of endogenous antioxidant defenses (e.g., increased antioxidant enzyme activity and Hsp70/90 expression), and improvement of BTB integrity (61, 88). In some cases, the protective effects are associated with the regulation of intestinal microbiota, as observed with phloridzin and dognoside (89). Ultimately, these interventions help restore testosterone levels, promote spermatogenesis, and improve testicular tissue structure.

### 5.4 New nanotechnology treatments

Selenium plays a critical role in healthy spermatogenesis, testicular development, and sperm motility. Selenium nanoparticles (SeNPs), functionalized with active targeting ligands, exhibit excellent biocompatibility and enable efficient, targeted delivery of therapeutic agents (90). Recent studies have demonstrated that the combined administration of SeNPs and metformin confers protective effects against streptozotocin-induced testicular oxidative damage in diabetic rats. This protective mechanism involves the suppression of redox imbalance, improvement of sperm quality, and upregulation of nuclear factor erythroid 2–related factor 2 (Nrf2) expression (91). Similarly, zinc oxide nanoparticles (ZnO NPs) have been shown to exert protective effects against diabetes-induced testicular damage. These effects are attributed not only to the potent antioxidant and hypoglycemic properties of ZnO NPs, but also to their ability to downregulate pro-apoptotic gene expression and upregulate the expression of steroidogenesis-related regulatory genes within testicular tissue (92, 93). Collectively, selenium and zinc oxide nanoparticles demonstrate promising protective effects against diabetes-induced testicular oxidative damage and sperm dysfunction. Experimental evidence supports the role of these nanoparticles in improving sperm quality, highlighting their value as adjunctive agents in the management of male reproductive disorders associated with diabetes.

### 5.5 Other treatments

Montelukast, a cysteinyl leukotriene receptor 1 antagonist, has been shown to attenuate testicular injury through its anti-inflammatory, antioxidant, and autophagy-inducing properties (94). Similarly, bezafibrate, a peroxisome proliferator-activated receptor alpha (PPARα) agonist, has been shown to suppress inflammation and oxidative stress, thereby alleviating diabetic spermatogenesis disorder (95). Tropisetron, an antagonist of 5-Hydroxytryptamine (5-HT) type 3 receptor, has been demonstrated to reduce testicular inflammation induced by streptozotocin in diabetic rats. Furthermore, the late sodium current inhibitor ranolazine, as well as the mechanistic target of rapamycin (mTOR) inhibitor rapamycin and the chromium-based compound chromium picolinate, have demonstrated protective trends against diabetic testicular injury by inducing autophagy, inhibiting ER stress, oxidative damage, and apoptosis (96–99). Islet transplantation may also serve as an therapeutic strategy for diabetic complications in men by delaying diabetic testicular interstitial fibrosis (100, 101).

### 5.6 Combination treatments

Zinc combined with metformin restores zinc homeostasis by activating the PI3K/AKT/mTOR pathway, thereby enhancing steroidogenesis and improving semen quality in male mice with T2DM (102). Vitamin D enhances the efficacy of metformin in alleviating testicular dysfunction associated with T2DM (103). Co-administration of chrysin with MIRET significantly ameliorates diabetic testicular histopathological testicular histopathological and biochemical damage and diminishes pro-apoptotic effects (104). Cilostazol and sildenafil alleviate testicular damage in diabetes by exerting anti-inflammatory and antioxidant effects (105). The combination of sitagliptin and L-theanine demonstrates potential to significantly reduce diabetic testicular dysfunction, indicating a promising therapeutic approach (106).

### 5.7 Future perspectives

Although the aforementioned studies provide several potential therapeutic strategies for alleviating diabetic testicular dysfunction and impaired spermatogenesis, most of the current evidence is derived from animal models, with clinical data remaining relatively limited. Therefore, future research should focus on optimizing these interventions to enhance their clinical efficacy while minimizing potential side effects and safety concerns. Such efforts may contribute to the development of more personalized and targeted therapeutic approaches for diabetes-related complications, ultimately facilitating the clinical translation of these emerging treatments.

## 6 Discussion

DM is a metabolic disorder that induces multi-organ complications primarily through neuropathy and vascular damage, with significant adverse effects on the male reproductive system (107–109). Although the diabetes-induced male reproductive health remains incompletely understood, recent studies have highlighted diabetic testicular dysfunction, particularly in relation to impaired spermatogenesis and disruption of the testicular microenvironment. The pathogenesis of diabetes is primarily driven by hyperglycemia and insulin deficiency (82). Hyperglycemia disrupts the metabolic support provided by SC to developing germ cells, directly impairing sperm production and quality. Additionally, hyperglycemia results in reduced sperm count, abnormal sperm morphology, and a marked decline in sperm motility (14, 21).

Mitochondria play a vital role in providing energy for sperm motility. However, under diabetic conditions, mitochondrial dysfunction leads to excessive production of ROS, impaired ATP synthesis, and activation of mitochondria-dependent apoptotic pathways. This results in the apoptosis of germ cells and a decline in sperm parameters (110). Additionally, diabetes disrupts the tight junctions between Sertoli cells that constitute the BTB, compromising its integrity and permitting harmful systemic factors to infiltrate the seminiferous epithelium. This disruption impairs germ cell migration during spermatogenesis (52).

Endocrine dysregulation is another significant factor in diabetic testicular dysfunction. Diabetes affects the HPG axis, often leading to reduced levels of gonadotropins (LH, FSH) and testosterone (59). It also impairs the function of LC by suppressing the expression of steroidogenic enzymes such as steroidogenic acute regulatory protein (StAR), cholesterol side-chain cleavage enzyme (P450scc), and 3β-hydroxysteroid dehydrogenase (3β-HSD), ultimately reducing testosterone synthesis (111). The resulting low testosterone levels further exacerbate impairments in spermatogenesis and metabolic function, thereby perpetuating a vicious cycle (27).

**Figure 1:**
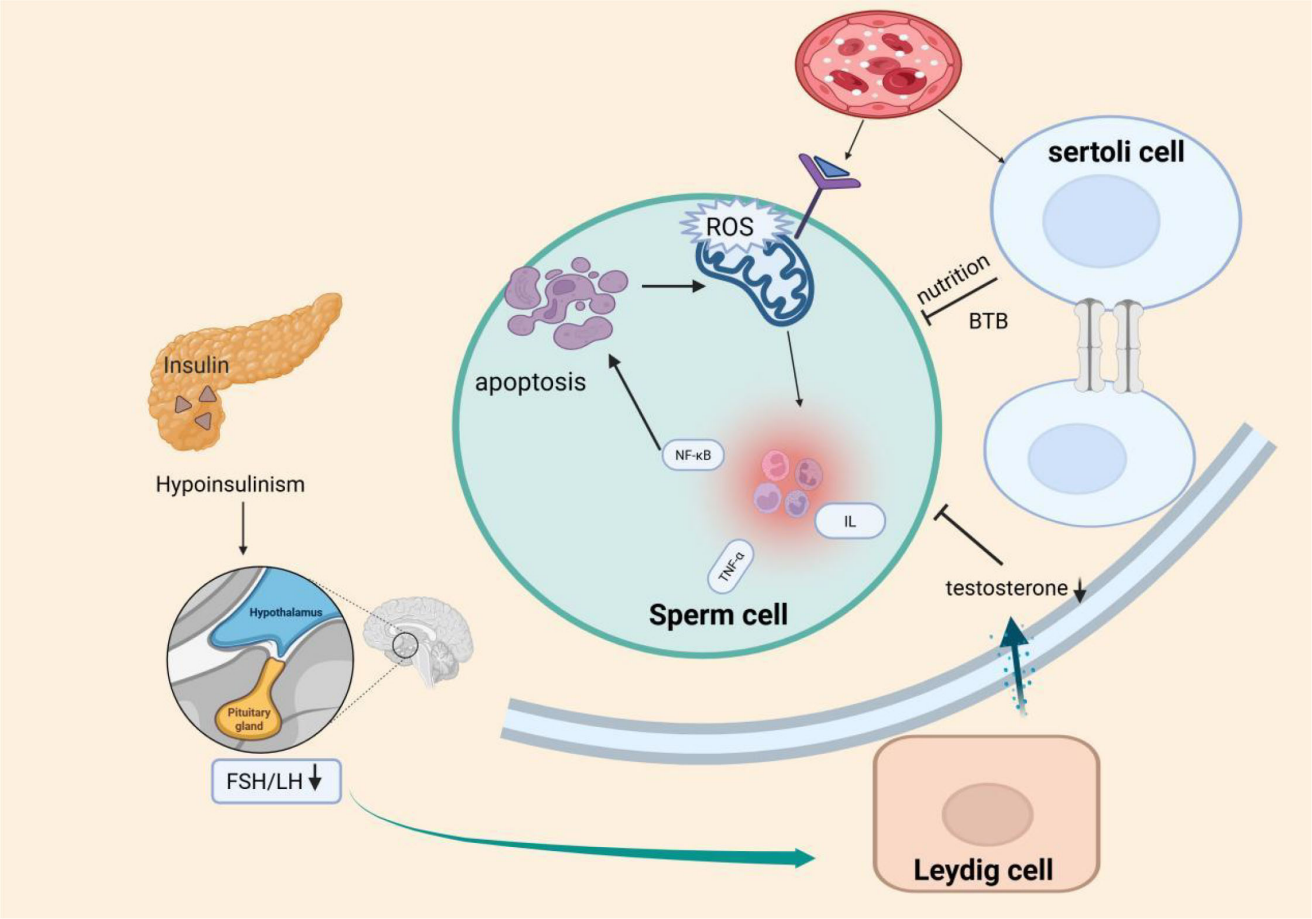
Proposed model of testicular injury and spermatogenic impairment induced by metabolic disturbances and insulin dysregulation in diabetes mellitus (DM).This schematic illustrates that hyperglycemia in DM primarily triggers oxidative stress, increased advanced glycation end products (AGEs), mitochondrial dysfunction, and inflammatory responses, ultimately disrupting the spermatogenic environment (e.g., blood-testis barrier impairment, BTB). Concurrently, insulin deficiency in diabetic patients impairs the hypothalamic-pituitary-gonadal (HPG) axis, reducing secretion of luteinizing hormone (LH) and testosterone (T). These factors may synergistically disrupt spermatogenesis, contributing to male infertility.

Diabetes negatively impacts testicular function through multiple mechanisms, including hyperglycemia, oxidative stress, accumulation of AGEs, inflammation, endocrine disruption, ER stress, and mitochondrial dysfunction (112). These pathological processes directly impair sperm quality, including concentration, motility, morphology, and DNA integrity, while also damaging the BTB, LC, and SC, thus disrupting the testicular spermatogenic microenvironment. Ultimately, these cascades lead to reduced male fertility or infertility (**Figure 1**).

While maintaining strict glycemic control remains critical, emerging therapeutic strategies targeting specific mechanisms of testicular damage offer promising avenues for enhancing reproductive health in diabetic men. These include the use of GLP-1 RA, Chinese Medicine, nanoparticles, probiotics, and targeted antioxidants and anti-inflammatory drugs. However, further research is required to evaluate the clinical translational potential and long-term safety of these novel interventions.
